# Tissue Regeneration and Remodeling in Rat Models after Application of *Hypericum perforatum* L. Extract-Loaded Bigels

**DOI:** 10.3390/gels10050341

**Published:** 2024-05-17

**Authors:** Yoana Sotirova, Yoana Kiselova-Kaneva, Deyana Vankova, Oskan Tasinov, Diana Ivanova, Hristo Popov, Minka Hristova, Krastena Nikolova, Velichka Andonova

**Affiliations:** 1 Department of Pharmaceutical Technologies, Faculty of Pharmacy, Medical University of Varna, 9000 Varna, Bulgaria; velichka.andonova@mu-varna.bg; 2 Department of Biochemistry, Molecular Medicine and Nutrigenomics, Faculty of Pharmacy, Medical University of Varna, 9000 Varna, Bulgariaoskan.tasinov@gmail.com (O.T.); divanova@mu-varna.bg (D.I.); 3 Department of General and Clinical Pathology, Forensic Medicine and Deontology, Faculty of Medicine, Medical University of Varna, 9000 Varna, Bulgaria; hristo.popov@mu-varna.bg; 4 Department of Physiology and Pathophysiology, Faculty of Medicine, Medical University of Varna, 9000 Varna, Bulgaria; hristova_minka@mu-varna.bg; 5 Department of Physics and Biophysics, Faculty of Pharmacy, Medical University of Varna, 9000 Varna, Bulgaria; krastena.nikolova@mu-varna.bg

**Keywords:** hyperforin, inflammation, St. John’s wort, TNF-α, wound healing

## Abstract

The wound-healing effect of St. John’s Wort (SJW) is mainly attributed to hyperforin (HP), but its low stability restricts its topical administration. This study investigates how “free” HP-rich SJW extract (incorporated into a bigel; B/SJW) and extract “protected” by nanostructured lipid carriers (also included in a biphasic semisolid; B/NLC-SJW) affect tissue regeneration in a rat skin excision wound model. Wound diameter, histological changes, and tissue gene expression levels of fibronectin (Fn), matrix metalloproteinase 8 (MMP8), and tumor necrosis factor-alpha (TNF-α) were employed to quantify the healing progress. A significant wound size reduction was achieved after applying both extract-containing semisolids, but after a 21-day application period, the smallest wound size was observed in the B/NLC-SJW-treated animals. However, the inflammatory response was affected more favorably by the bigel containing the “free” SJW extract, as evidenced by histological studies. Moreover, after the application of B/SJW, the expression of Fn, MMP8, and TNF-α was significantly higher than in the positive control. In conclusion, both bigel formulations exhibited beneficial effects on wound healing in rat skin, but B/SJW affected skin restoration processes in a comprehensive and more efficient way.

## 1. Introduction

The healing of a wound is an intricate process characterized by dynamic changes in the wound microenvironment and directed toward restoring tissue integrity and functional capacity following injury. The physiological wound-healing process is usually divided into four consecutive and overlapping phases: hemostasis, inflammation, proliferation, and remodeling [1]. These must all occur in a specific sequence, at a specific time, and for a precise amount of time for an efficient and timely repair process. Impaired coordination and regulation delay wound healing and can lead to detrimental outcomes, such as the formation of an increased scar, a non-healing wound, or a risk of infection. Therefore, although there is overlap in the phases, each phase is necessary for the next to be completed [2].

Hemostasis is the first stage of wound healing. When the skin is wounded, the immediate response is to stop the bleeding, which is achieved by platelet activation, vasoconstriction, and the formation of a blood clot [3].

Inflammation is an important part of the wound-healing response. Acute inflammation is the first tissue response to wounding, and a cascade of activation or mobilization of more cell types leads to the swift formation of a proinflammatory microenvironment [3,4]. Wound healing, whether sterile or not, is accompanied by an inflammatory reaction [3,5]. Polymorphonuclear (neutrophils (Neu), eosinophils (Eos)) and mononuclear (monocytes, macrophages (Ma), lymphocytes (Ly), and plasmacytes (Pl)) leukocytes are the cells that, together, orchestrate the inflammatory response. They perform complex interactions and precisely coordinate each moment of the resolution of inflammation [3].

Tumor necrosis factor-alpha (TNF-α), a proinflammatory factor involved in the inflammatory phase of wound healing, is secreted by Neu and Ma infiltrating the wound [6]. In patients treated with inhibitors of TNF-α, skin recovery was postponed [7]. In an animal model of mouse skin wound healing, TNF-α concentration levels were measured in the homogenate supernatants of the collected wounds at various time intervals. The TNF-α cytokine was detected just after wound creation, and the concentration rapidly increased during the first several hours. The peak concentration level was reached on day 1, and levels declined thereafter to the basal level [8]. Topical treatment with saponin from *Panax notoginseng* (Notoginsenoside Ft1) reduced the infiltration of monocytes and suppressed the TNF-α proinflammatory cytokine in diabetic wound beds. Accordingly, the mRNA expression levels of TNF-α were significantly decreased in the skin of diabetic animals at day seven post-injury [9].

Proliferation aims to reduce the area of the wound and relatively recuperate skin structure and functions. In this phase, various extracellular matrix proteins are secreted to replace the fibrin clot; they also produce collagen to build a stable platform for subsequent neoangiogenesis and re-epithelialization [10]. Among these polypeptides, fibronectin (Fn) stands out with its crucial role in the wound healing mechanism, including the initial injury response to tissue repair and remodeling [11,12,13]. Fn is a multifunctional protein that plays a central role in wound healing by regulating cell adhesion, migration, extracellular matrix formation, growth factor signaling, angiogenesis, wound contraction, and inflammation [14,15,16,17]. Its intricate involvement in these processes highlights its significance as a key mediator of tissue repair and regeneration.

The last phase, remodeling, includes the transformation of the deposited proteins, epithelialization, and scar tissue maturation in order to restore the initial tissue organization to the extent possible [10]. Matrix metalloproteinases (MMPs) are a big family of endoproteases that are involved in the many processes requiring extracellular matrix remodeling and degradation. MMPs play a role in cells’ proliferation, differentiation, and apoptosis, as well as in cell and tissue repair. Variations in MMPs expression levels are observed in wound healing [18]. MMP8 is the most abundant collagenase in many wounds, and it degrades collagen types I, VII, VIII, and X. The wound infiltration with Neu results in the production and secretion of various factors, including MMP-8 [19]. The selective inhibition of MMP-8 caused delayed wound healing and incomplete re-epithelialization in diabetic mice [20].

Manifold studies have highlighted the prospects of wound management using medicinal plants. For instance, *Hypericum perforatum* L., also known as St. John’s Wort (SJW), is an herb well known in the folk medicine of the Balkans, Malaysia, and China [21,22,23,24]. It is mainly used to treat gastrointestinal infections, gastric ulcers, diabetes mellitus, common cold, jaundice, hepatic and biliary disorders, various neurological disorders, including depression and sciatica [22,23,25,26], and skin problems like wounds, burns, and bruises [25,26,27]. Scientific studies are focusing mainly on the antidepressant [28,29], antimicrobial [30], and wound healing potential [31,32] of the plant extracts.

The wound-healing potential of *H. perforatum* has been attributed to its main bioactive compounds, including hyperforin (HP), hypericin, isoquercitrin, hyperoside, epicatechin, quercetin, and rutin, which promote proliferation, migration, increases in the production and activation of fibroblast collagen cells, and angiogenesis, which explains the wound-healing effect of this plant [33,34,35]. The same studies discuss these bioactive compounds as the main ones possessing well-known anti-inflammatory and antioxidant activities, which contribute additionally to the wound-healing effect of the herb [33].

HP is known to be one of the main compounds “in charge” of SJW’s wound-healing effect. However, the phytochemical in question is highly reactive to light and oxygen [36,37]; hence, its topical administration is hindered. This setback could be feasibly solved by entrapping HP in nanostructured lipid carriers (NLCs)—a strategy widely applied for the dermal delivery of labile lipophilic compounds [38,39,40]. NLCs possess a biocompatible and biodegradable profile and superb skin tolerability while providing improved drug stability and enhanced skin hydration [41,42,43]. These features highlight their suitability as a delivery platform for topical wound treatment and have increased their employment as such in recent years. Various studies prove that NLCs are able to accelerate wound healing by enhancing the therapeutic effects of many active pharmaceutical ingredients: from synthetic molecules such as atorvastatin and allopurinol [44,45] to different phytoconstituents—curcumin and resveratrol, ferulic acid and *Lavandula officinalis* essential oil, and thymoquinone [46,47,48].

The discussed nanovehicles are commonly obtained as low-viscosity aqueous dispersions. However, they can be further incorporated into manifold semisolid formulations to prolong skin contact time. Regarding ease of application, patient acceptability, and enhanced drug permeation, bigels may be a particularly appropriate choice [49]. Therefore, in terms of topical drug delivery, a synergistic effect is expected when combining biphasic gels and NLCs.

Our previous works demonstrated the elaboration of stable, non-toxic bigels containing HP-rich SJW extract: “unprotected” (B/SJW) and incorporated into NLCs (B/NLC-SJW) [50,51]. The current study aims to investigate their effect on regenerative processes in rat skin using an excision wound model.

## 2. Results

### 2.1. Wound Healing

The influence of time and type of the applied semisolid on the size of the wounds was analyzed by a two-way analysis of variance and Duncan’s test to determine significant differences. The results are presented in Table 1, where wound size is given as the mean diameter of seven measurements and the calculated standard deviation (SD) in the negative control group (G0), the positive control group where a commercial wound-healing product was applied (G1), the group treated with bigel containing “free” SJW extract rich in HP (B/SJW; G2), and the group treated with bigel comprising nanoencapsulated HP-rich SJW extract (B/NLC-SJW; G3). Wound diameter was recorded at the 2nd, 7th, 14th, and 21st days (D2, D7, D14, and D21, respectively) of the treatment.

On the second day of the research period, the most efficient therapeutic effect was observed in animals treated with SJW wort extract-containing formulations (Table 1). On the last day (D21), the smallest wound size was observed in animals from the G3 group (1.500 ± 0.548 mm). These observations could be associated with the positive effect of B/NLC-SJW on tissue recovery, previously demonstrated in an incisional wound model in rats [50].

**Figure 1:**
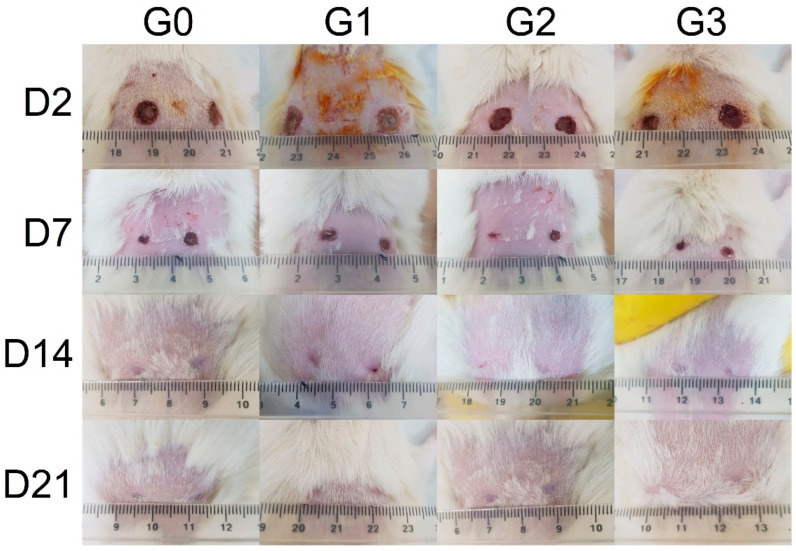
Wound repair in the studied groups (G0–G3) at the respective post-excision days (D2–D21).

The progression of wound healing in the experimental animals is presented in Figure 1. After 21 days, complete repair was achieved in all studied groups. However, recovery was seen to occur faster in the animals treated with HP-rich SJW extract-based formulations, even after 2 days of application.

### 2.2. Histological Studies

The histopathological changes in the different time ranges have similar morphological changes, and no significant dynamics in the number of inflammatory cells were observed during the different periods of granulation tissue maturation in the studied groups. The maturation of granulation tissue conditionally passes through three periods: young granulation tissue (rich in Neu), granulation tissue in the stage of organization (rich in Ma, Ly, and Pl), and mature connective tissue (cicatrix poor in inflammatory cells).

**Figure 2:**
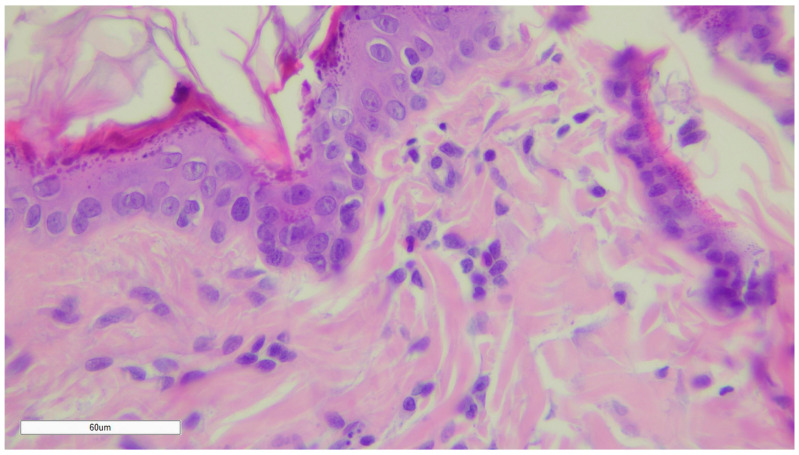
Healthy (unwounded) rat skin. Magnification ×400.

As a control group (given in Figure 2), we took skin from rats without a wound defect (normal skin).

**Figure 3:**
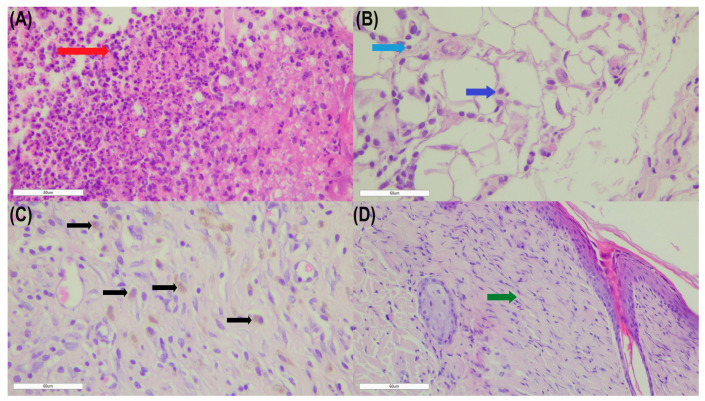
Wound healing at different observation periods: D2 (**A**), D7 (**B**), D14 (**C**), and D21 (**D**). High neutrophil (Neu) count (red arrow) can be seen after a 2-day application period (**A**). Lymphocytes (Ly) (light blue arrow) and plasmacytes (Pl) (blue arrow) predominate after 7 days of treatment (**B**) while macrophages (Ma) (black arrows), Ly, and Pl are prevalent at D14 (**C**). Scant inflammatory infiltration and advanced collagenization (green arrow) are observed after the 21-day application period (**D**). Magnification ×400.

Figure 3 represents the wound healing in the different observation periods. The presented photos are of the animals from G2, as the changes there are most visible, and healing (scarring) has occurred at the fastest rate.

**Figure 4:**
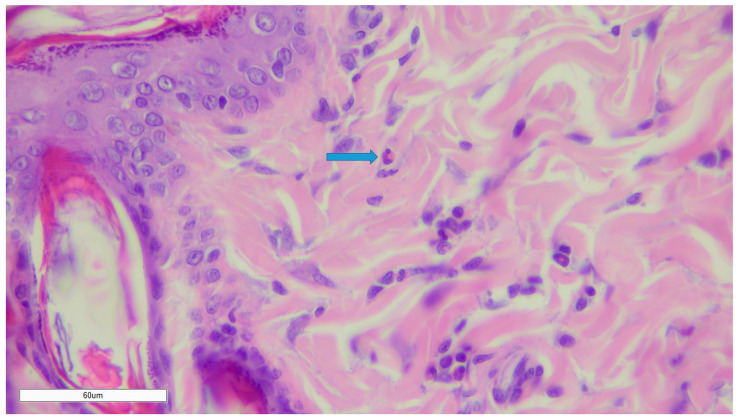
Eosinophil (Eos) in the dermis. Magnification ×400.

After 21-day treatment, according to experimental work and data acquisition, a tendency to increase eosinophilic leukocytes in the superficial dermis was observed only in G3 (Figure 4).

Table 2 presents the results of the levels of statistical significance of the differences obtained after applying unifactorial and multifactorial dispersion analyses, reflecting the influence of each factor: time and type of used product.

The influence of the used product is important for the amount of Eos, Ma, and Ly. It was found that the combination of the two factors had a statistically significant effect only on Ma.

The measured number of cells and results from Duncan’s test are given in Table 3. The presence of a variety of ranks proves the high degree of variation in the indicators’ values and proves the significant influence of the studied factors on the investigated parameters.

### 2.3. Gene Expression

**Figure 5:**
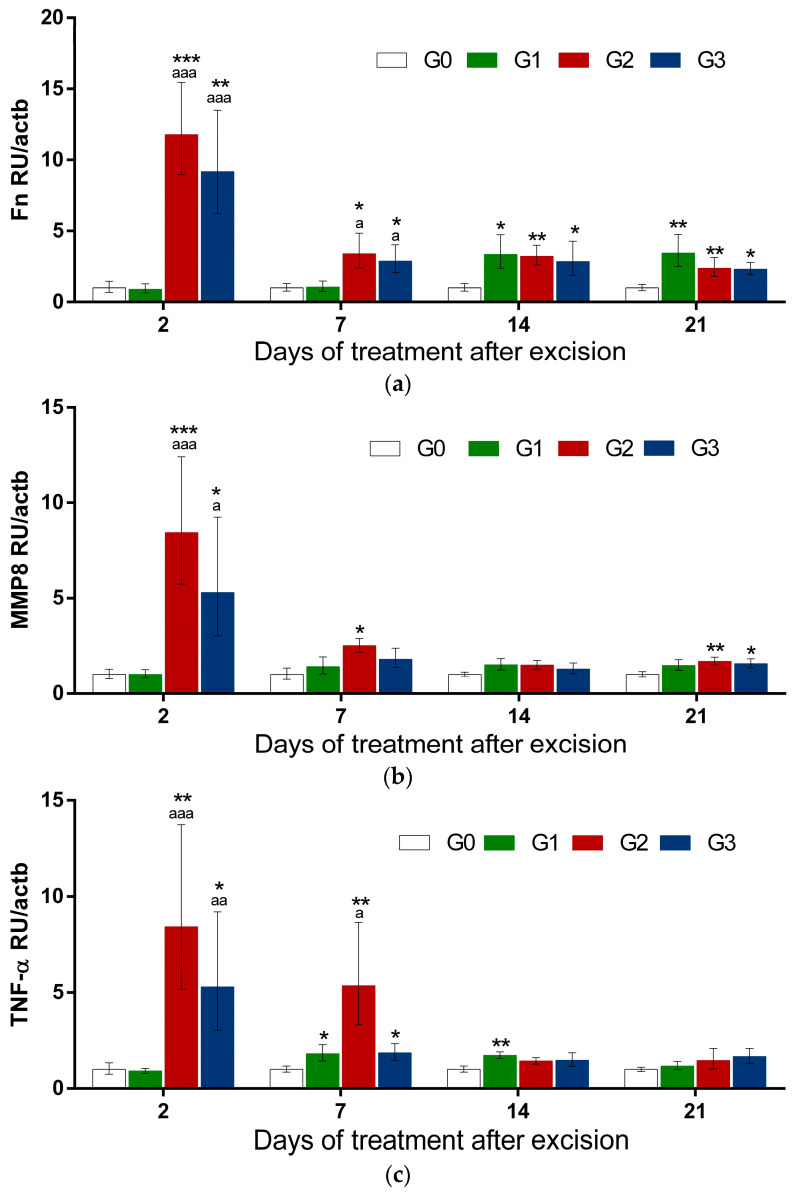
Expression levels of fibronectin (Fn), matrix metalloproteinase 8 (MMP8), and tumor necrosis factor alpha (TNF-α) in rat skin in a model on wound injury: (**a**) Fn mRNA-relative levels; (**b**) MMP8 mRNA-relative levels; (**c**) TNF-α mRNA-relative levels. Results are presented as relative units (RU) divided by each sample beta-actin (actb) expression. * vs. G0; a vs. G1.

The expression levels of the following genes were analyzed so as to detect tissue regenerative and/or reorganizing processes in injured rat skin: Fn, MMP8 (collagenase), and TNF-α. Data are presented in Figure 5.

Both B/SJW (G2) and B/NLC-SJW (G3) formulations application caused a remarkable increase in mRNA levels for all of the studied genes already on the 2nd day after their application (Figure 5a–c). The fold change in Fn was more than 11 (*p* < 0.001) and more than 8 for MMP8 (*p* < 0.001) and TNF-α (*p* < 0.01) in the G2 group as compared to the injured skin without other treatments (G0). Similarly, in G3, Fn was up-regulated more than nine times (*p* < 0.01), and MMP8 and TNF-α were up-regulated more than five times (*p* < 0.05). For the three studied genes, expression in G3 was lower than in G2 at D2 and D7, but the difference was without statistical significance. However, in both G2 and G3, the expression of Fn, MMP8, and TNF-α was significantly higher than in G1 (treated with a commercial product), while the expression at D2 did not change. Similarly, at D7, Fn was significantly higher in G2 (vs. G1) for Fn and TNF-α and in G3 for Fn. mRNA levels for the three genes gradually decreased over time. However, Fn remained significantly higher in G2 and G3 than in G0 for all next treatment periods (7, 14, and 21). The same tendency was observed for MMP8 and TNF-α expression, which was normalized in G2 and G3 as compared to the G0 and/or G1 groups at D14 and D21. In comparison, commercial product (G1) resulted in a slight but significant increase in TNF-α at D7 and D14 after treatment, whereas MMP8 remained without change in this group.

## 3. Discussion

*H. perforatum* preparations are used in folk medicine for the treatment of skin inflammation and to stimulate wound healing [31,32,35]. A lot of research has been focused on its properties related to tissue regeneration. For example, oily extracts containing *H. perforatum* have been found to stimulate healing of surgical wounds in women with scars from caesarean sections and episiotomy by increasing the epithelial reconstruction and reducing inflammation, itching, and redness [52,53,54]. In patients with plaque-type psoriasis, the topical application of SJW ointment reduces TNF-α concentrations in the dermis, endothelial, and dendrite cells, which may be one of the mechanisms for the stimulation of lesions healing [55,56]. Also, the topical application of *H. perforatum* extract accelerated wound healing by stimulating re-epithelialization and collagen accumulation in linear incisions in rats [32]; stimulated epithelialization and hydroxyproline synthesis in oral mucosa surgical wounds of rats with induced diabetes mellitus, causing faster healing [57]; promoted epidermal thickness and the number of blood vessels that formed in a rat model of thermal burns [58]; and stimulated fibroblasts collagen production and the promotion of fibroblast polygonal shape that are necessary for wound repair by closing damaged area in chicken fibroblast cells [31]. Additionally, the oral administration of an SJW tincture increased wound healing in incisions, excisions, and dead space wounds in rat models [59].

Despite well-recognized results of traditional therapy practice, experimental wound models [31,32,57,59], and clinical studies [52,53,54], the molecular mechanisms of the wound-healing activity of *H. perforatum* L. are still not well understood.

As already stated, HP in SJW, the principal “carrier” of the plant’s wound-healing activity, is very susceptible to environmental factors. For that purpose, in primo loco, we prepared twenty blank NLC samples via sequential emulsification, high-shear homogenization, and ultrasonication. We employed different lipids (solids: beeswax or glyceryl behenate (GB); liquids: almond oil or borage oil (BO)) and variable processing parameters (homogenization speed, ultrasonication time, and temperature). After a thorough characterization, only two models based on GB were considered acceptable [60].

**Figure 6:**
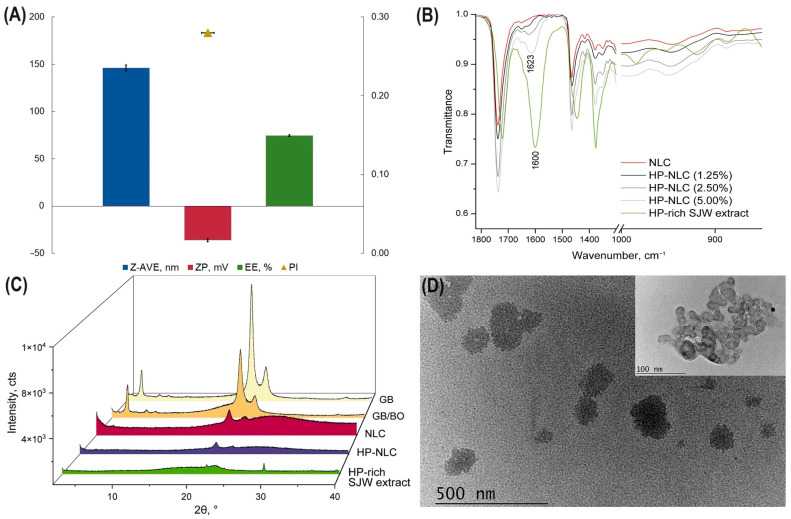
Characterization of the nanostructured lipid carrier (HP-NLC) dispersion loaded with SJW extract, rich in HP: mean size (Z-AVE), polydispersity index (PI), zeta potential (ZP), and entrapment efficiency (EE) of HP-NLC (**A**); attenuated total reflectance Fourier-transform infrared (ATR-FTIR) spectra of HP-rich SJW extract (green line), blank nanostructured lipid carriers (NLC; red line), and extract-loaded nanoparticles (greyscale lines) (**B**); X-ray diffraction (XRD) patterns of glyceryl behenate (GB), lipid blends (GB and borage oil (GB/BO)), nanoparticulate dispersions (NLC and HP-NLC), and HP-rich SJW extract (**C**); transmission electron microscopy (TEM) images of HP-NLC (**D**).

Next, we obtained an SJW extract through modified maceration (conducted in the dark in an argon ambiance) with an HP yield of almost 45%. The latter was incorporated into the selected blank NLC samples (in a concentration of 1.25 *w*/*w*%). The model based on GB and BO (HP-NLC) was selected as the preferable one on the grounds of the following observations (Figure 6) [50]:

- The HP-NLC sample possessed particles with a mean size in the nanoscale (below 200 nm) and high zeta potential values (>|30| mV). The system presented a relatively uniform size distribution expressed as a polydispersity index lower than 0.3 (Figure 6A);
- The successful entrapment of the HP-rich SJW extract was proven through quantitative HPLC analysis: the encapsulation efficiency was nearly 75% (Figure 6A). ATR-FTIR studies were used as qualitative proof—the absorption band with a maximum of 1623 cm^−1^, present in the HP-NLCs (1.25%), was found to be characteristic of the SJW extract itself (Figure 4). In addition, by preparing overloaded samples (HP-NLC (2.50%) and HP-NLC (5.00%)), the suitability of the selected extract concentration was confirmed;
- The disorder in the inner matrix of HP-NLCs was demonstrated by their X-ray diffraction pattern (Figure 6C). The absence of the extract’s characteristic reflections in the diffractogram suggested its presence in a non-crystalline state within the nanoparticles;
- The carriers’ inner morphology was also investigated by TEM (Figure 6D), and particles with imperfect structures and delimited solid lipid grains were observed [50].

The low viscosity of the HP-NLC dispersion necessitated its “conversion” into a semisolid formulation prior to topical application. To achieve this aim, Poloxamer 407/BO-based bigels with different hydrogel-to-oleogel proportions (from 90:10 to 60:40) were prepared and studied. HP-NLCs were incorporated into the hydrophilic phase of the bigel (B/NLC-SJW), providing a 0.5% (*w*/*w*) extract concentration in the semisolid. To investigate the influence of the colloidal carriers on the therapeutic potential of HP-rich SJW extract, a biphasic gel containing “unprotected” extract (in the same final concentration yet incorporated into the oleogel) was also obtained (B/SJW) [50].

The utilization of semisolids comprising 80% hydrogel and 20% organogel was based on their:

- Shear-thinning behavior (preferable during topical application);
- Favorable mechanical properties, i.e., spreadability, firmness, cohesiveness, and adhesiveness;
- Skin-tolerable pH value;
- Physical stability under stress conditions [50].

All these results demonstrate the successful development of semisolid formulations in terms of their technological characteristics. To confirm and compare the wound-healing effect, we conducted an in vivo experiment involving an excision wound rat model. The degree of wound contraction, the dynamics in the count of different inflammatory cells (Neu, Eos, Ma, Ly, and Pl) during the treatment, and the levels of Fn, MMP8, and TNF-α were employed as measures of therapeutic activity. A commercial herbal cream with an anticipated therapeutic outcome was selected as the positive control, and the negative control was represented by non-treated wounded animals.

The beneficial effect of HP-rich SJW extract-containing formulations was demonstrated when comparing the wound sizes within the experimental groups in the different periods studied. Through this study, B/NLC-SJW showed a superb therapeutic potential—the smallest wound area after 21 days of treatment was observed in G3. In order to confirm the obtained macroscopic data, we investigated the wound healing process on a tissue level.

Neu were the dominant cell type in all studied groups at day 2 after excision. At day 7, Neu were still the predominant granulocytes in the tissue samples of the G1, G2, and G3 groups, while Ly dominated the cellular infiltrate of the G0. The finding of granulocytic leukocytes (Neu and Eos) at the wound edge was well-defined in the G2 and G3 groups on day 2. On the other hand, the results show the presence of a more pronounced Ma and Neu infiltration in the G1 and G2 groups at the first two time points (D2 and D7). These data suggest the presence of a more pronounced inflammatory response in the tested groups.

We presume that the pronounced cellular infiltration by granulocytes and Ma, independent of differences in the distribution pattern, indicates an ongoing autolytic debridement. The latter suggests that the tested substances provide suitable conditions for a functioning immune system to selectively remove devitalized tissues [61]. Although conservative, this natural process removes the necrotic or infected tissue that may prolong the inflammatory phase and impede wound contraction as well as re-epithelialization [61,62].

Dynamic changes in the cellular infiltrate were observed on day 14. Cell infiltration was reduced in wound tissues on G1 and G2 compared to groups on days 2 and 7 post-incision. Unlike these, an increase in the total cell infiltrate was observed in the G0 and G3 groups compared to that on day 7. In the G0 group as a whole, despite the presence of an increased number of Neu compared to the 7-day samples, mononuclear cells Ly and Ma, as well as Pl, presented dominantly. In the G1, G2, and G3 groups, mononuclear cells dominate again, but against the background of a tendency to decrease granulocytes.

We assume that this distribution pattern of the cell populations is most likely due to the presence of cells of the M2 Ma phenotype, which, together with the other cells, are engaged in the proliferative and remodeling processes of the tissues [2,63]. The dominant presence of Ly in all tissue samples is also evidence of the resolution of the acute inflammatory process after excision. This pattern of cell distribution was emphasized in the G2 group, in which, in addition, cell infiltration in the wound tissue was lowest compared to the other groups.

Interest represents the G3, in which the total cell infiltrate is highest compared to the other groups at 14 days. Here, in the background of Neu decreasing, all other cells (Ma, Ly, and Pl) are at peak levels as compared to previous periods for the group as well as to the other groups of 14 days. This increased cell number is the cause of the observed maximum level of cell infiltration in G3 at day 14.

We assume that the increased Ma infiltration in this group is a response to the obvious slower decline of Neu at day 7 and ongoing efferocytosis in tissues [2,4]. On the other hand, we can also assume the parallel presence of the M2 reparative phenotype [2,63].

Ly is the dominant cell type at day 14. This finding suggests that changes in the microenvironmental conditions have occurred that stimulate to a greater degree the involvement of the adaptive immune response in the modulation of inflammation and the later stages of early repair [64,65]. Ly infiltrate is also sure evidence of successful resolution of inflammation in all studied groups [63].

On day 21, only Eos is present, a finding observed in healthy and uninjured skin.

In summary, a marked decrease in total cell infiltrate was observed in G1 and especially G2 compared to G0 and G3 at 14 days. The histological finding showed that the count of granulocytes and mononuclear cells decreased linearly throughout the entire period studied in the G2 group. This trend is also observed to a known extent in G1. Therefore, we hypothesize that the process of wound healing proceeds more regularly and smoothly in the G2 group.

Data show that the application of both B/SJW (G2) and B/NLC-SJW (G3) formulations already caused a significant increase in expression levels for all of the studied genes at D2 after their application (Figure 5a–c), where the fold change in Fn was more than 11 (*p* < 0.001) and more than 8 for MMP8 (*p* < 0.001) and TNF-α (*p* < 0.01) in the G2 group as compared to the injured skins without other treatment (G0). Similarly, in G3, Fn was up-regulated more than nine times (*p* < 0.01), and MMP8 and TNF-α were up-regulated more than five times (*p* < 0.05).

Once the skin injury appears, the cutaneous wound immediately enters the first stage of hemostasis, as blood vessel constriction prevents excessive bleeding and platelet aggregation forms a plug. The formation of a blood clot serves as a provisional matrix for cell recruiting as well [66]. The surrounding cells release inflammatory cytokines and growth factors in order to attract Ma, Neu, and Ly, which defend the wound site from infectious agents [67]. Ma is responsible for the production of reactive oxygen species (ROS), matrix metalloproteinases (MMPs), platelet-derived growth factor (PDGF), transforming growth factor-β (TGF-β), vascular endothelial growth factor (VEGF), and fibroblast growth factor (FGF) [68]. On the other hand, ROS play a stimulatory effect on MMP production in the wounds [69]. Neu produces Fn, a protein that has a structural function, mediates interactions among the components of the extracellular matrix (ECM), and plays a role as a bridge between cells [70,71]. The next wound repair stage includes the processes of granulation and angiogenesis. Newly formed blood vessels donate oxygen to wound tissue, which is important for wound healing. Ma is involved in the degradation of blood clots and the production of cytokines and chemokines, which recruit fibroblasts to the wound site [69]. The established significantly elevated mRNA levels of Fn and MMP8 in *H. perforatum* formulations (G2 and G3) are consistent with the application of this herb for wound healing. The observed boosted expression of the respective genes could be an explanation of its regenerative properties.

Fn is a high-molecular-weight dimeric glycoprotein of the extracellular matrix [72]. It is actively engaged in wound healing as it works together with various proteins in the wound bed and plays an important role in adhesion as part of the extracellular matrix. Two types of Fn are recognized in vertebrates: soluble plasma Fn and insoluble cellular Fn [17].

Hypericin and HP, the main constituents of *H. perforatum* extract, increase the mRNA levels of Fn in the parental cell line, while protein levels are reduced in HT-29 adenocarcinoma cells [73] and in the human hepatoma cell line [74].

Normally, MMPs expression in normal, healthy skin is generally low. When the skin wound happens, the up-regulation and expression of many MMPs have been described, including MMP8 [20]. MMPs are a family of calcium-dependent, zinc-containing endopeptidases involved in extracellular matrix remodeling and degradation [18]. MMP8 is a collagenase, mainly expressed by Neu, and has as substrates collagen I, II, and III; aggrecan; serpins; and 2-MG [75]. Thus, the overexpression of MMP8 upon *H. perforatum* formulations may be related to their regenerative effects, contributing to more effective skin remodeling. MMP-8 activity was slightly up-regulated after seven days from the infliction of the wound and has been established to have a beneficial role in wound healing, as it might be the response to the healing process in the body. In support of this hypothesis, the selective inhibition of MMP8 caused delayed wound healing and incomplete re-epithelialization in diabetic mice [20]. In addition, MMP8-deficient mice exhibited a significant lag in wound healing, caused by a delay in Neu infiltration, inflammation, and impaired re-epithelialization [76]. The active recombinant MMP8 topical application on diabetic wounds demonstrated the speed up of significant wound healing and skin re-epithelialization [77].

*H. perforatum* treatment reduces plasma MMP8 and MMP9 levels and capillary destruction in a rat model of skin thermal burns [78].

In the inflammatory phase of wound healing, the recruited Neu and Ma produce TNF-α, thus extending the inflammatory response [6]. In addition to its pro-inflammatory function, TNF-α cytokine regulates the activity of cells such as fibroblasts, vascular endothelial cells, and keratinocytes, which are involved in wound healing [79]. Thus, the established pronounced up-regulation of TNF-α at D2 and D7 in the G2 and G3 groups could rather be related to its participation in skin healing processes, as TNF-α in G0 was significantly lower than in the G2 and G3 groups. In support of this finding is a study where *H. perforatum* oil-loaded nanofiber dressings reduced the oxidative stress index and slightly increased the TNF-α level in rats with diabetic wounds [80].

The difference between the effects of G2 and G3 on the expression of Fn, MMP8, and TNF-α at D2 and D7 was not significant. However, the tendency for mRNA levels to be lower in G3 as compared to G2 could be due to the nanoparticle formulation, suggesting the slower release of the active ingredients. Fn, MMP8, and TNF-α expression levels were significantly higher in the G2 and G3 groups as compared to the commercial product (G1) at D2 and for Fn at D7, demonstrating the high effectiveness of the studied B/SJW and B/NLC-SJW formulations regarding their application for skin wound healing.

The favorable effect of the studied *H. perforatum* formulations on wound healing was best demonstrated by the wound size data (Table 1). The application of both SJW-containing bigels resulted in smaller wounds than those measured in G0 and G1 in all studied periods. Moreover, the highest wound contraction after the 21-day treatment period was observed in the G3 group. Despite the reported slightly lower degree of wound contraction, the wound-healing effect of B/SJW was significantly more pronounced when taking into account histological changes and regenerative markers values. A possible explanation of these results is the ability of NLCs not only to preserve the entrapped active ingredient(s) but also to modify their delivery. The nanosystems in question can provide drug release in a slower, controlled manner without rapid expulsion [81,82]. Of course, steady drug liberation will affect the values of the inflammatory cells and highly sensitive markers (Fn, MMP8, and TNF-α) to a lesser extent.

## 4. Conclusions

In our study, we investigated the wound-healing effects of two bigels comprising HP-rich SJW extract (B/SJW and B/NLC-SJW). Both semisolids, containing either “free” extract or extract encapsulated in NLCs, appear to promote tissue regeneration and recovery in a rat skin excision model. Despite the symbiosis between bigels and lipid nanoparticles as carriers in the skin-healing formulation design, B/SJW more effectively affects separate phases of tissue repair by modulating both the innate immune response and non-specific cell protection, as well as the adaptive response. The application of the biphasic gel containing “free” SJW extract also led to a significant increase in mRNA levels for all studied genes (i.e., Fn, MMP8, and TNF-α), thus supporting the histological data obtained. Assuming all the obtained results, it can be concluded that despite the successful preservation of HP in SJW by NLCs, B/SJW can “manage” wound healing in a more effective manner.

## 5. Materials and Methods

### 5.1. Materials

Ketamine 5% (Bremer Pharma GmbH, Warburg, Germany), xylazine 2% (Alfasan Int., Woerden, The Netherlands), Jodseptadon 10% (Chemax Pharma Ltd., Sofia, Bulgaria), and sodium chloride 0.9% solution (B. Braun Melsungen AG, Melsungen, Germany) were of pharmaceutical grade. TRItidy G reagent (AppliChem GmbH, Darmstadt, Germany), RevertAid First Strand cDNA Synthesis Kit (ThermoScientific, Waltham, MA, USA), and Kapa Sybr fast qPCR kit (Kapa Biosystems, Wilmington, MA, USA) were used in the gene expression analyses. 

B/SJW and B/NLC-SJW were obtained as previously reported by us [50]. The preparation of HP-rich SJW extract, its inclusion in NLCs, and the formulation of bigels as their semisolid platform are thoroughly described in our previous works and briefly discussed in Section 3.

### 5.2. Methods

#### 5.2.1. Experimental Animals

The protocol of the study was approved by the Commission for Ethical Treatment of Animals at the Bulgarian Food Safety Agency (permit number: 265/02.06.2020). All experiments were under the EU Directive 2010/63/EU for animal experiments, the Basel Declaration, and the International Council for Laboratory Animal Science ethical guidelines for researchers [83,84,85].

One hundred twelve male Wistar rats (*Rattus norvegicus albinus*), weighing 200 ÷ 250 g each, were provided by the Vivarium of the Medical University of Varna. They were situated individually in standard plastic cages at 22 ± 1 °C, at about 55% relative humidity, and in a 12L:12D light cycle. They were fed a standard pellet diet and had unlimited access to drinking water during the whole experiment.

#### 5.2.2. Wound Excision Model

The wound excision model was used to study the wound-healing effect of formulated bigels in rats. Prior to injury, rats were anesthetized by the intramuscular injection of 35.0 mg/kg ketamine (5%) and 5.0 mg/kg xylazine (2%). The dorsal hair of each rat was shaved and disinfected with povidone–iodine (10% cutaneous solution). Two full-thickness excisional wounds were created using a 6 mm biopsy punch, diametrically opposite to the dorsal line of the animals.

For the experimental procedure, the animals were randomly assigned into the following four groups (*n* = 28), and the test formulations were administered topically, once daily:

- Group G0: wound group, untreated, negative control group;
- Group G1: a positive group treated with a commercial herbal semisolid formulation used for wound healing, containing extracts from *Aloe vera*, *Prunus amygdalus*, *Vitex negundo*, and *Rubia cordifolia*;
- Group G2: a group treated with B/SJW;
- Group G3: a group treated with B/NLC-SJW.

Animals from each group were euthanized on the 2nd, 7th, 14th, and 21st post-operative days with an overdose of anesthetic (ketamine and xylazine in doses of 70.0 mg/kg and 15 mg/kg, respectively). The animals’ deaths were confirmed by cardiopulmonary arrest, a drop in body temperature, and the absence of hind leg reflexes [86]. Skin samples from each animal were stored for subsequent molecular biological analyses.

#### 5.2.3. Wound Area Measurements

Complete and permanent wound closure was a main criterion for the indication of progressive healing. Wound diameter (mm) was employed as the manner to quantify the healing progress and was recorded at the indicated time points [87,88].

#### 5.2.4. Histological Examinations

The studies were performed on biopsy materials taken from the skin of experimental animals at different time ranges. Each sample was fixed in 10% neutral buffered formalin for 18–24 h, then released for standard processing and embedding in melting point paraffin 52–54 °C in order to prepare paraffin blocks. Four micron-thick sections were prepared from the paraffin blocks and stained with standard hematoxylin–eosin to assess histological changes in each wound. All observations were performed using an Olympus BX50 light microscope (Olympus Optical Co LTD, Melville, NY, USA). The enumeration of inflammatory cells at different time ranges in the area of the wound defect was performed at 10 fields of magnification HPF (×400).

Different inflammatory cells (Neu, Ly, Ma, Pl, and Eos) were quantified, and the obtained data were statistically processed (described in Section 5.2.6).

#### 5.2.5. Gene Expression Analyses

Total RNA extraction was performed as follows: approximately 30 mm^3^ (20 mg) of frozen skin tissue were smashed immediately in 500 μL TRItidy G reagent (AppliChem GmbH, Darmstadt, Germany) in 1.5 sterile tubes. The next steps followed the protocol as described by manufacturer. Isolated RNA was reversely transcribed with RevertAid First Strand cDNA Synthesis Kit (Thermo Fisher Scientific, Waltham, MA, USA) using (dT)18 primer. Reverse transcription reaction was performed according to the manufacturer’s protocol at a final volume of 10 μL. Gene expression analysis was performed using qReal-Time PCR method with Kapa Sybr fast qPCR kit (Kapa Biosystems, Wilmington, MA, USA) on a QuantStudio™ 5 Real-Time PCR System instrument (Applied Biosystems, Thermo Fisher Scientific, Waltham, MA, USA). Actin beta (Actb) was used as endogenous control. Primer sets were as follows: Actb F: GGGAAATCGTGCGTGACATT; Actb R: GCGGCAGTGGCCATCTC; Fn1 F: TATGAGAAGCCTGGATCCCCT; Fn1 R: TGAAGATTGGGGTGTGGAAGG; MMP8 F: ACTTCTTCGTAAACAACCAATGCTG; MMP8 R: GGTCCACTGAAGAAGAGGAAGAA; TNF-α F: GACCCTCACACTCAGATCATCTTCT; TNF-α R: TGCTACGACGTGGGCTACG.

Gene expression levels were calculated according the 2^−ΔΔCt^ method [33] and were presented in relative units (RU) as compared to a control group (G0), where expression is considered to be equal to 1.

#### 5.2.6. Statistical Analysis

Data from seven wound area measurements were processed to obtain the mean value and SD. An analysis of variance was conducted to assess the influence of the type of formulation applied, time, and the combination of these factors on wound healing (wound size and inflammatory cells count). Duncan’s test for multiple comparisons was used to determine significant differences in the wound area and polymorphonuclear and mononuclear cells, with a significance level of *p* < 0.05. Statistical analyses were performed using SPSS ver. 26.0 software (IBM Corp., Armonk, NY, USA).

Student’s *t*-test was used to compare two independent datasets in the gene expression analysis using GraphPad Prism ver. 6 software (GraphPad Software Inc., San Diego, CA, USA).

## Figures and Tables

**Table 1:** Two-way analysis of variance on the effect of time and type of the used product on the healing wounds (mean ± SD, *n* = 7). The group of untreated, wounded animals (negative control) is given as G0, the group of animals treated with a commercial herbal formulation is G1, the group treated with bigel containing “free” St. John’s wort (SJW) extract rich in hyperforin (HP) (B/SJW) is G2, and the group treated with bigel comprising nanoencapsulated HP-rich SJW extract (B/NLC-SJW) is G3. Measurements were taken after the 2-, 7-, 14, and 21-day application periods (D2, D7, D14, and D21) of the respective formulations.

	Wound Diameter, mm			
Time, Days	G0	G1	G2	G3
0	6.000 ± 0.00 ^a^	6.000 ± 0.00 ^a^	6.000 ± 0.00 ^a^	6.000 ± 0.00 ^a^
2	5.250 ± 0.27 ^b^	4.667 ± 0.52 ^c^	3.917 ± 0.59 ^d^	3.750 ± 0.52 ^d,e^
7	4.750 ± 0.27 ^b,c^	3.583 ± 0.80 ^d,e,f^	3.333 ± 0.26 ^d,e,f,g^	3.000 ± 0.45 ^f,g,h^
14	4.683 ± 0.42 ^c^	3.568 ± 0.49 ^d,e,f^	3.287 ± 0.18 ^e,f,g^	2.435 ± 0.18 ^h^
21	2.833 ± 0.52 ^g,h^	3.167 ± 0.98 ^e,f,g^	2.583 ± 0.59 ^h^	1.500 ± 0.55 ^i^

The means values with the same letters do not differ significantly at *p* ≤ 0.05 as analyzed by Duncan’s test.

**Table 2:** Influence of the application time and applied product on Neu, Eos, Ma, Ly, and Pl cells.

Factor	Neu	Eos	Ma	Ly	Pl
Time	0.000	0.003	0.000	0.000	0.000
Product	0.166	0.008	0.000	0.019	0.131
Time × Product	0.391	0.750	0.002	0.196	0.209

**Table 3:** Values of inflammatory cells in the studied groups at the respective post-excision day.

Group	Time	Neu	Eos	Ma	Ly	Pl
G0	D2	45.67 ± 8.76 ^c,d^	1.33 ± 0.33 ^b,c^	2.00 ± 0.58 ^b,c^	18.33 ± 8.82 ^a^	1.67 ± 0.88 ^a,b^
	D7	1.67 ± 0.85 ^g^	3.00 ± 1.78 ^b,c^	1.33 ± 0.47 ^c,d^	10.67 ± 1.89 ^b,c^	0.67 ± 0.47 ^b,c^
	D14	6.67 ± 5.18 ^f,g^	0.00 ± 0.00 ^c^	5.33 ± 3.53 ^b^	8.00 ± 3.06 ^b,c^	2.33 ± 0.88 ^a^
	D21	0.00 ± 0.00 ^g^	2.00 ± 0.00 ^b,c^	0.00 ± 0.00 ^d^	0.00 ± 0.00 ^d^	0.00 ± 0.00 ^c^
G1	D2	54.67 ± 5.61 ^b,c^	2.67 ± 1.76 ^b^	8.67 ± 2.03 ^a^	14.33 ± 2.73 ^a,b^	1.33 ± 0.67 ^a,b,c^
	D7	21.33 ± 17.33 ^e^	0.33 ± 0.33 ^c^	9.00 ± 3.61 ^a^	8.67 ± 3.28 ^b,c^	2.33 ± 1.45 ^a^
	D14	6.00 ± 5.51 ^f,g^	1.00 ± 0.58 ^c^	5.00 ± 1.00 ^b,c^	10.67 ± 3.71 ^b,c^	1.33 ± 0.67 ^a,b,c^
	D21	0.00 ± 0.00 ^g^	0.00 ± 0.00 ^c^	0.00 ± 0.00 ^d^	0.00 ± 0.00 ^e^	0.00 ± 0.00 ^c^
G2	D2	70.67 ± 5.42 ^a^	6.33 ± 4.13 ^a,b^	5.00 ± 0.41 ^b,c^	10.00 ± 1.41 ^b,c^	2.33 ± 0.26 ^a^
	D7	33.33 ± 11.60 ^d,e^	2.00 ± 1.16 ^b,c^	4.67 ± 0.88 ^b,c^	12.00 ± 0.58 ^a,b,c^	1.00 ± 0.00 ^a,b,c^
	D14	1.67 ± 0.88 ^g^	0.33 ± 0.33 ^c^	2.33 ± 1.45 ^b,c,d^	8.67 ± 2.03 ^b,c^	0.67 ± 0.67 ^b,c^
	D21	0.00 ± 0.00 ^g^	0.67 ± 0.33 ^c^	0.00 ± 0.00 ^d^	0.00 ± 0.00 ^d^	0.00 ± 0.00 ^c^
G3	D2	64.33 ± 8.17 ^a,b^	9.67 ± 5.24 ^a^	1.67 ± 1.20 ^b,c,d^	6.00 ± 0.58 ^c,d^	1.00 ± 0.58 ^a,b,c^
	D7	13.33 ± 12.35 ^f,g^	3.00 ± 2.08 ^b,c^	1.33 ± 0.33 ^c,d^	8.00 ± 2.52 ^b,c^	0.67 ± 0.33 ^b,c^
	D14	10.00 ± 5.00 ^f,g^	0.00 ± 0.00 ^c^	10.67 ± 2.33 ^a^	14.67 ± 0.33 ^a,b^	1.67 ± 0.67 ^a,b^
	D21	0.00 ± 0.00 ^g^	3.33 ± 0.47 ^b,c^	0.00 ± 0.00 ^d^	0.00 ± 0.00 ^d^	0.00 ± 0.00 ^c^

Means in a column with a common superscript letter (^a–g^) differ (*p* < 0.05) as analyzed by Duncan’s test.

## Data Availability

The data presented in this study are available on request from the corresponding authors.
